# CircRNA-1806 Decreases T Cell Apoptosis and Prolongs Survival of Mice After Cryptococcal Infection by Sponging miRNA-126

**DOI:** 10.3389/fmicb.2020.596440

**Published:** 2020-11-13

**Authors:** Lei Zhang, Keming Zhang, Wenjie Fang, Hang Li, Yingfang Li, Weiwei Jiang, Dongying Hu, Carolina Coelho, Xiaogang Liu, Liangqi Cai, Wanqing Liao, Weihua Pan

**Affiliations:** ^1^Department of Dermatology and Venereology, Changzheng Hospital, Second Military Medical University, Shanghai, China; ^2^Shanghai Key Laboratory of Molecular Medical Mycology, Shanghai Institute of Medical Mycology, Changzheng Hospital, Second Military Medical University, Shanghai, China; ^3^MRC Centre for Medical Mycology, University of Exeter, Exeter, United Kingdom; ^4^Department of Dermatology, The First Affiliated Hospital of Xiamen University, Fujian, China

**Keywords:** cryptococcal infection, circular RNA, micro-RNA, cell cycle, cell apoptosis

## Abstract

CircRNAs are a recently well-known regulator that mediates a variety of biological processes. Cryptococcus neoformans is an environmental fungal pathogen that can cause fatal cryptococcal meningitis in immunocompromised individuals. However, the involvement of circRNA in cryptococcal infection remains unclear. In this study, high-throughput microarray was performed to identify the circRNA expression profile in cryptococcal meningitis patients. Circ_0001806 was significantly decreased in cryptococcal meningitis individuals. Then the effects of circ_0001806 and its interaction with miRNAs were explored *in vivo* and *in vitro*. The knock-down of circ_0001806 led to higher fungal infection and shorter survival in an experimental murine cryptococcosis model. Transcriptome analysis showed that decreased circ_0001806 regulated pathways related to the host antimicrobe response in T cells. Furthermore, *in vitro* experiments showed that circ_0001806 positively modulates ADM level, decreasing cell apoptosis and G1S arrest in T cells. Finally, we found circ_0001806 exerted its effects by sponging miRNA-126 in T cells. Taken together, our results reveal the role of circRNA-1806/miRNA-126 in the regulation of cell cycle and apoptosis in cryptococcal infection and can provide a new insights of the pathogenesis of cryptococcal infection.

## Introduction

Cryptococcal meningitis (CM) is a serious central nervous system infectious disease, accounting for approximately 15% of the world’s AIDS-related mortality, and the main pathogen is *Cryptococcus neoformans* (*C. neoformans*) (Rajasingham et al., 2017). Although HIV-associated cryptococcal meningitis ranks the most common cause of meningitis in most areas of the world, HIV-negative cryptococcal meningitis remains a neglected issue considering the rising number of individuals undergoing transplant and with cell-immunity defects (Williamson et al., 2017). In the immunocompetent host, *C. neoformans* mainly causes asymptomatic subclinical infection or latent infection, supported by an epidemiological result that most people have a history of exposure to *Cryptococcus* during childhood (Goldman et al., 2001). In patients with deficient immune responses, *Cryptococcus* is usually fatal due to its strong neurotropism (Vu et al., 2014). As a mycosis caused by the opportunistic fungus, the progression and outcome of cryptococcosis mainly depends on the interplay between the host’s immune response and fungal pathogens.

Over the past few years, circRNAs, characterized by the absence of the classical 5′-PolyA or 3′-caps and resistance to the degradation role of RNase, have been identified as a new class of epigenetic regulators in a variety of biological processes from aging to tissue development to cancer (Qu et al., 2017). Evidence from recent years has shown that circRNAs are involved in many immune responses by epigenetic modifications in a cell-specific manner (Chen X. et al., 2019). Aberrant circRNA expression profiles have also been identified in many immune-related disease, such as psoriasis (Qiao et al., 2018). Given the importance of the immune system in defense against pathogens, many circRNAs are reported to be associated with the host response against viruses (Cadena and Hur, 2017), bacteria (Huang et al., 2017), parasites (Ren et al., 2018). However, no studies have investigated whether and how circRNAs are involved in medical mycosis as host regulators.

Given the important regulatory role of circRNAs in the host immune response, we sought to identify the involvement and functional role of circRNAs in HIV-negative cryptococcal meningitis patients. First, circRNA microarray and quantitative real-time PCR were performed to identify the aberrant circRNA profile in CM patients. Then, the involvement of circRNA in the progression of cryptococcal infection was examined in an experimental cryptococcosis murine model. Following the investigation of the impact of circRNA on the T cell transcriptome after C. neoformans exposure, the molecular mechanism was also investigated. Here, we report decreased circ_0001806 aggregated Cryptococcal infection by impairing the T cell response via miRNA-126 sponges in CM patients.

## Materials and Methods

### PBMCs Isolation

Five milliliters of venous blood was obtained from patients with cryptococcal meningitis who were admitted to Shanghai Changzheng and Shanghai Changhai hospital. Buffy coat from healthy donors was obtained from the blood bank of PLA in Shanghai. Then, PBMCs were isolated by Ficoll-10771 (Sigma Aldrich, United States) according to the manufacturer’s manual. All samples were stored at −80°C before experiments. The clinical characteristics of patients with cryptococcal meningitis are provided in Supplementary Table 1. This study was approved by the Ethics Committee of Changzheng Hospital. Consent was obtained from all participants.

### *C. neoformans* Strain

The C. neoformans strain H99 (serotype A) used in this study was a gift from J. Perfect (Duke University, United States). The fresh H99 isolate was cultured in YPD liquid medium with moderate shaking for 48 h to reach the stationary phase. Heat-killed H99 strain (prepared in a 65°C water bath for 30 min) was used unless otherwise noted.

### CircRNA Microarray and Analysis

The circRNA commercial kit (4 × 180K, Human Circular RNA Microarray; SBC, Shanghai) was used to identify the differentially expressed circRNA profile in PBMCs from both patients with cryptococcal meningitis and healthy donors. Total RNA was extracted and purified using a miRNeasy Mini Kit (QIAGEN, Germany) and checked for RIN number to inspect RNA integration by an Agilent Bioanalyzer 2100 (Agilent Technologies, United States). Then, qualified total RNA (RIN ≥ 7.0 and 28S/18S ≥ 0.7) was amplified and labeled by a Low Input Quick Amp WT Labeling Kit (Agilent Technologies, United States). Labeled cRNA was purified by an RNeasy mini kit (QIAGEN, Germany). Each slide was hybridized with 1.65 μg Cy3-labeled cRNA using a Gene Expression Hybridization Kit (Agilent Technologies, United States). After 17 h of hybridization, slides were washed in staining dishes (Thermo Shandon, United States) with a Gene Expression Wash Buffer Kit (Agilent Technologies, United States). Slides were scanned by an Agilent Microarray Scanner with default settings, dye channel: green, scan resolution = 3 μm, PMT 100%, 20 bit. Data were extracted with Feature Extraction software 10.7 (Agilent Technologies, Santa Clara, CA, United States). Raw data were normalized by a quantile algorithm with the limma packages in R. GO and KEGG analyses of circRNA host genes were performed according to the methods previously described (Kanehisa et al., 2004).

### Quantitative Real-Time PCR

Total RNA was extracted using TRIzol (Invitrogen, United States), and the quality and quantity were detected by a NanoDrop 2000. Reverse transcription reactions were performed using a PrimeScript RT reagent kit (Takara, Japan). The SYBR Green method was used for quantitative real-time PCR. For the detection of circRNAs, backspliced circRNA sequences were targeted to design divergent primers based on the sequence in the circRNA database^1^. All the primer sequences are provided in Supplementary Table 6.

### Experimental Cryptococcosis Murine Experiments

All experiments with animals were performed in the Animal Facility of Second Military Medical University. Six- to eight-week-old C57BL/6 female mice were raised under routine conditions. H99 was grown for 18 h at 37°C while shaking at 180 rpm in YPD broth. Cells were washed with PBS and resuspended in 2.5 × 10^7^ cells/ml PBS. The counterpart circRNA in mice was identified as circ_0000008^1^, and the cholesterol-modified siRNA was synthesized by Sangon, Shanghai (Sequence is provided in Supplementary Table 3). One day before infection, 10 OD cholesterol-modified siRNA and control siRNA were intraperitoneally injected. Then, the mice were anesthetized with 2% isoflurane anesthesia followed by intranasal infection of 20 μl fresh H99 suspension. Two weeks after infection, lung and brain tissue were isolated. To test the knock-down efficiency, total RNAs was extracted from mice lung and brains tissues, and quantitative PCR was performed. To count CFU, the homogenates were suspended in sterile water with serial dilution. A 50 μl suspension was added to each YPD plate and then incubated at 30°C for 2 days. Histopathological analysis was performed by HE & PAS staining, and slides were observed by optical microscopy (Nikon Eclipse E100, Japan). H&E histology scores, as described previously, were performed by two independent colleagues blinded to the protocol for metrics of inflammation (Jawhara and Poulain, 2007). All animal experiments were performed according to the National Institute of Health Guide for the Care and Use of Laboratory Animals and approved by the Scientific Investigation Board of Second Military Medical University (Approval number: NSFC-31770161).

### The Subcellular Identification of Circ_0001806

PBMCs from the buffy coat of healthy donors were obtained using Ficoll-10771 followed by microbead isolation. In short, T cells, monocytes and B cells were isolated using CD3, CD14, and CD19 microbead kits (Miltenyi, Germany), respectively, according to the manufacturer’s protocol. Then, the cytoplasm and nuclear components of CD3^+^ T cells were extracted using a Paris^TM^ kit (Thermo Fisher, United States). The expression of circ_0001806 was detected using SYBR Green-based qRT-PCR as previously described.

### Cell Culture Transfection and Infection

Jurkat T cells were cultured in 10% FBS RPMI 1640 in a 5% CO_2_ incubator, as routine. The has_circ_0001806 siRNA, hsa-miR-126-3p mimic and hsa-miR-126-3p inhibitor were synthesized by Sangon Biotech, Shanghai (sequences are provided in Supplementary Table 7). The circ_0001806 overexpressed plasmid was constructed using GV485 vecter and synthesized by Genechem, Shanghai. Lipofectamine RNAiMAX (Thermo Fisher, United States) was used to deliver siRNA or plasmid to Jurkat T cells in accordance with the manufacturer’s manual. In short, 5 × 10^5^ Jurkat T cells were plated in a 12-well plate. Then, siRNA-iMax or plasmid-iMax was prepared and added to each well at a final concentration of 60 pmol. After 48 h of transfection, heat-killed *C. neoformans* cells were added at an MOI of 5 to 1 (five fungal cells to one Jurkat T cell), followed by functional analysis.

### Apoptosis and Cell Cycle Analysis

Apoptosis and cell cycle detection were performed using a BD Accuri C6 flow cytometer. In brief, cell suspensions were collected and stained with Annexin V-FITC/PI and PI cell cycle kits (Fuyuanbio, China) in accordance with the manufacturer’s instructions. In short, for apoptosis, the cell suspension was adjusted to 1 × 10^6^/ml, and 0.2 ml suspension was centrifuged followed by resuspension using PBS. After removing all the medium, the pellet was resuspended in staining buffer, and 5 μl Annexin-FITC and PI were added. Following 10–15 min incubation protected from light at room temperature, the apoptosis rate was detected (Ex = 488 nm, Em = 530 nm). For cell cycle analysis, the cell pellet was resuspended in −20°C 75% ethanol and fixed overnight at 4°C. After washing with PBS, the cell pellet was stained with 0.5 ml PI and RNasseA buffer and incubated for 30 min protected from light at 37°C followed by cell cycle detection (Ex = 488 nm).

### Transcriptome Analysis

After infection, triplicate cell suspensions were collected, and RNA was extracted as previously described. Circ_0001806 transcriptome detection was performed on the BGISEQ-500 platform to generate approximately 11.01 Gb per sample in total. In short, low quality, adaptor-polluted and unknown base reads were removed before downstream analysis. Clean reads were used to map to the reference genome using HISAT. Fold change ≥ 2 and P value < 0.001 were used as cut-offs to identify the differentially expressed genes. GO classification and KEGG pathway analyses were also performed. Differentially expressed genes that encode transcription factors (TFs) in plant and animal research were also predicted.

### MicroRNA Pull-Down (RIP)

All the following experiments were performed as described previously (Su et al., 2015). In brief, cells were transfected with a biotin-tagged specific probe or control probe and harvested after 24 h of transfection. Cells were crosslinked by formaldehyde, equilibrated in glycine buffer and scraped with lysis buffer. Cell samples were sonicated and centrifuged. Then, 50 μl of the supernatant was saved as input analysis. The supernatant lysate was incubated with M-280 beads (Thermo Fisher, United States) followed by washing and incubation to reverse the formaldehyde cross-links. Subsequently, RNAs were repurified by TRIzol. Purified mRNA and miRNAs were detected by qRT-PCR assay using an All-in-One miRNA qRT-PCR Detection Kit (GeneCopoeia, United States) according to the manufacturer’s instructions. All the probes used are listed in Supplementary Table 8.

### RNA Fluorescence *in situ* Hybridization

RNA fluorescence *in situ* hybridization was performed as described (Han et al., 2017). Cells were prepared in 6-well plates and incubated at 37°C in a 5% CO_2_ incubator until 50%–60% confluency. Cell supernatant was aspirated, and paraformaldehyde was used to fix cells for 15–20 min. Prehybridization (1 × PBS/0.5% Triton X-100) was added, and the cells were hybridized in buffer with DIG-labeled probes and incubated at 60°C overnight followed by washing with SSC. DAPI was added, and the cells were protected from light for 8 min. Signals were detected using an upright microscope (Nikon, Japan). The parameters were as follows: blue channel, Ex = 330–380 nm, Em = 420 nm; green channel, Ex = 465–495 nm, Em = 515–555 nm; red channel, Ex = 510–560 nm, Em = 590 nm. The primers are listed in Supplementary Table 9.

### Luciferase Reporter Assay

The luciferase reporter construct was developed by cloning the human ADM 3′-UTR sequence into the pMIR-Report construct (Ambion, United States) via *Sac*I and *Hin*dIII sites. All primers are listed in Supplementary Table 10. Briefly, HEK-293 cells were plated in a 48-well plate and co-transfected with 50 nM miRNA mimics or negative control oligonucleotides (Sangon, China), 200 ng of firefly luciferase reporter and 50 ng of pRL-TK (Promega, United States) using the JetPRIME reagent (Polyplus, United States). Cells were collected 36 h after the last transfection and analyzed using the Dual-Luciferase Reporter Assay System (Promega, United States).

### Western Blot Analysis

Total protein was extracted using RIPA premixed with proteinase inhibitor. The quantity was measured by using a micro BCA kit (Thermo Fisher, United States). β-actin was used as an internal control. The antibodies for ADM and GAPDH were ADM polyclonal antibody (ABclonal, United States) and GAPDH monoclonal antibody (ABclonal, United States), respectively. In short, samples were adjusted to the same concentration, mixed with loading buffer and then subjected to electrophoresis using NuPAGE (Thermo Fisher, United States) at an initial voltage of 45 V followed by 65 V. After transferring and blocking, primary antibody was added to each membrane overnight incubation at 4°C. After secondary antibody incubation, an enhanced chemiluminescence kit (Thermo Fisher, United States) was used to detect the bands as specified by the manufacturer. The relative protein level was calculated as follows: grayscale value of target protein/grayscale value of control.

### Statistical Analysis

Data are reported as the means from at least three independent experiments. Continuous variables were displayed as the mean ± standard deviation and compared by Student’s t test or one-way ANOVA analysis. The survival difference was determined using the log-rank test. *P* values of <0.05 were considered statistically significant. All statistical analyses were performed with GraphPad Prism software (La Jolla, CA, United States).

### Data and Materials Availability

All relevant data supporting the findings of this study are available in this article and its Supplementary Information files or from the corresponding author upon reasonable request.

## Results

### Reduced Circ_0001806 Levels Were Identified in PBMCs From CM Patients

**FIGURE 1:**
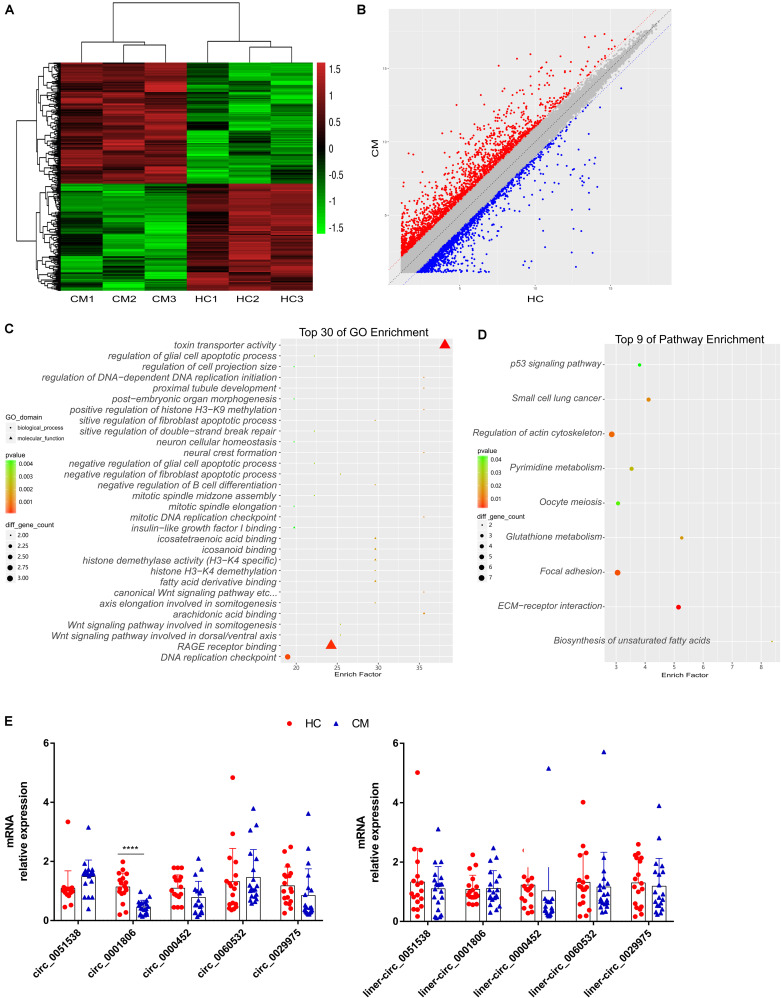
Reduced circ_0001806 levels were identified in PBMCs from cryptococcal meningitis patients, compared to healthy individuals. **(A)** Heat map of the differentially expressed circRNA; red corresponds to high expression, and green corresponds to low expression. Shown is data from three randomly selected individuals per group, from twenty CM individuals and eighteen healthy controls. **(B)** Scatter plot of the differentially expressed circRNA profile; red corresponds to high expression, and corresponds to for low expression. Shown is data from three randomly selected individuals per group, from twenty CM individuals and eighteen healthy controls. **(C)** GO analysis of differentially expressed circRNA host genes. **(D)** KEGG analysis of the differentially expressed circRNA host genes. **(E)** Expression levels of five circRNAs (Left) and their liner counterparts (Right) was verified by real-time PCR in PBMCs from twenty CM patients and eighteen healthy individuals. CM: Cryptococcus meningitis; HC: healthy controls. Unpaired *t*-test was used to calculate *p*-values, ****P < 0.0001.

Circular RNA microarray analysis was performed on PBMCs from three HIV-negative CM patients (non-organ transplant) and three healthy donors (the clinical characteristics are shown in Supplementary Table 1, No. P1-P3 were used in the microarray analysis). A dysregulated circRNA profile was identified in the PBMCs of CM patients. In total, compared with healthy individuals, using a cut-off value of *P* < 0.05 and a fold change factor ≥ 2, there were four hundred differentially expressed circRNAs in the PBMCs of CM patients, with 212 circRNAs upregulated and 188 downregulated (the profile of differentially expressed circRNAs is listed in Supplementary Table 2) (Figures 1A,B). After mapping to the hg19 human genome (the most heavily annotated and most commonly used reference database), 176 circRNAs were from positive-strand, and 223 were from negative-strand (hsa_circ_0007081_M_L was not identified). GO and KEGG analyses of differentially expressed genes circRNA host genes showed that them to be involved in DNA replication checkpoint, toxin transporter activity and RAGE receptor binding (Figure 1C). The majority of differentially expressed host genes according to KEGG were involved in the regulation of actin cytoskeleton and focal adhesion, and immune-associated pathways, such as the p53 signaling pathway, were also observed (Figure 1D). To validate our exploratory analysis, we selected five differentially expressed circRNAs, and verified their levels in PBMCs from twenty CM patients and eighteen healthy individuals (the clinical characteristics are shown in Supplementary Table 1). Compared with the healthy control group, circ_0001806 was significantly decreased in PBMCs from CM patients (Figure 1E, Left). As endogenous circRNAs may have similar functions to their liner counterparts, the linear counterpart levels were also verified in PBMCs from the same populations, but no significant difference in expression levels of linear counterparts were observed (Figure 1E, Right). The significant decrease in the level of circ_0001806, and not in its linear counterpart, indicates that a lower level of circ_0001806 was associated with CM patients.

### Decreased Circ_0001806 Contributed to Higher Fungal Burden at an Early Phase and Shorter Survival in Experimental Cryptococcosis

**FIGURE 2:**
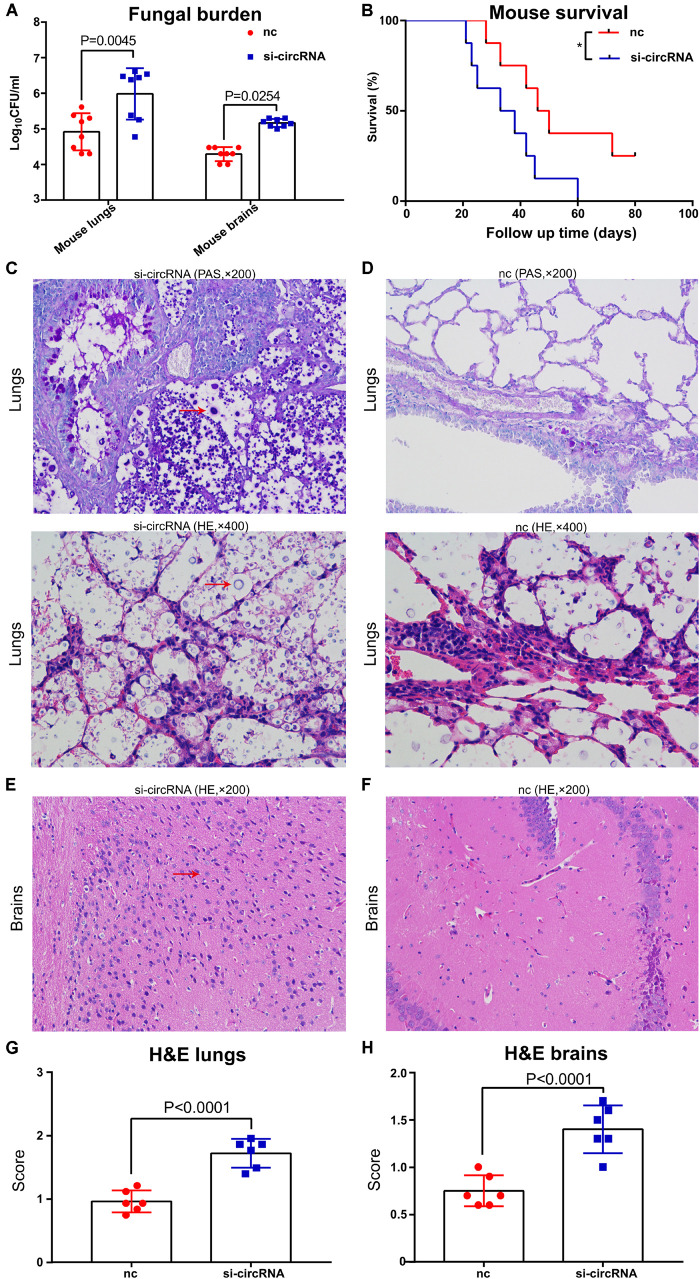
Circ_0001806 mouse counterpart siRNA contributed to the higher fungal burden and shorter survival in experimental murine cryptococcosis. Six- to eight-week-old C57BL/6 female mice were anesthetized with 2% isoflurane anesthesia followed by intranasal infection of 5 × 10^5^ fresh H99 cells to establish experimental cryptococcosis model. One day before infection, 10 OD cholesterol-modified circ_0001806 mouse counterpart siRNA and control siRNA were intraperitoneally (i.p.) injected. **(A)** Two weeks after infection, CFU of lungs and brains from eight mice transfected with circ_0001806 mouse counterpart siRNA and controls were determined. **(B)** The survival percentages of the siRNA group (blue squares) and the nc group (red squares) were tested. **(C–F)** Representative histopathological images of the lungs and the brains from the siRNA group **(C,E)** and the control group **(D,F)** using H&E and PAS staining. **(G,H)** H&E histological scores of the lungs **(G)** and the brains **(H)** from the siRNA group and the control group. Data are shown as the means ± SD from triplicate samples in groups of eight animals. Unpaired *t*-test was used to calculate *p*-values.

Then, we investigated the involvement of circ_0001806 in cryptococcal infection *in vivo*. C57BL/6 female mice were anesthetized with 2% isoflurane anesthesia followed by intranasal infection of 5 × 10^5^ fresh H99 cells to establish experimental cryptococcosis model. One day before infection, 10 OD cholesterol-modified circ_0001806 mouse counterpart siRNA and control siRNA (Sequence is provided in Supplementary Table 3) were intraperitoneally (i.p.) injected. The fungal burden and survival rate were observed after i.p. injection of the siRNA-circ_0001806 mouse counterpart. Compared with the control group, 14 days after infection, higher fungal burden in both the lungs and the brain was observed in the group with knockdown circ_0001806 (Figure 2A). In line with a previous study showing that the initial fungal burden after *C. neoformans* infection is usually linked to clinical outcome (Sabiiti et al., 2014), shorter survival was seen in the group with knockdown circ_0001806 (Figure 2B). The *in vivo* results were further supported by the hematoxylin-eosin (H&E) staining and periodic acid–Schiff (PAS) staining histopathological analysis (Figures 2C–F). Severe inflammation consisting of many *C. neoformans* was observed in both the lungs and brains of siRNA group (Figures 2C,E). In contrast, moderate inflammation consisting of a few *C. neoformans* was observed in both the lungs and brains of control mice (Figure 2D,F). In summary, decrease of circ_0001806 with siRNA lead to worsened cryptococcocal disease, indicating circ_0001806 was involved in the progression of cryptococcal infection in murine mice.

### Circ_0001806 Was Mainly Expressed in the Cytoplasm of CD3^+^ T Cells and Could Be Induced Following *C. neoformans* Exposure

**FIGURE 3:**
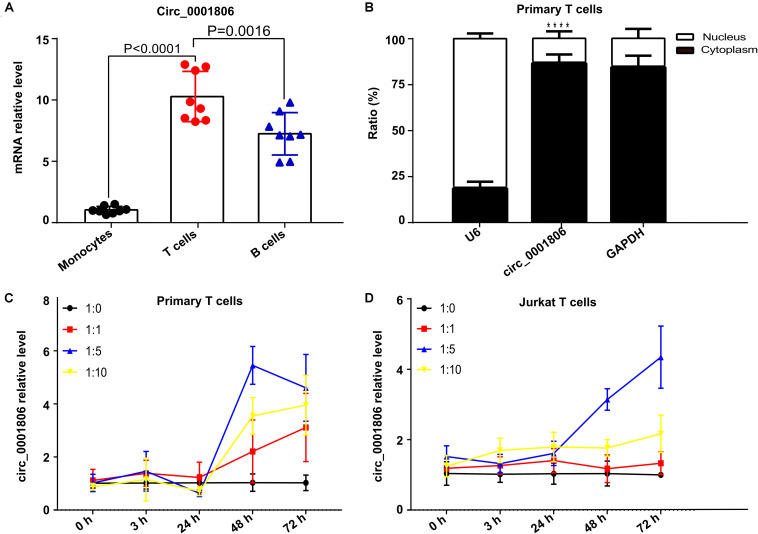
Circ_0001806 was mainly expressed in the cytoplasm of CD3^+^T cells and induced upon infection with *C. neoformans*. **(A)** Relative levels of circ_0001806 in peripheral blood primary CD14^+^monocytes, CD3^+^ T cells and CD19^+^B cells. Data shown are means ± SD from triplicate samples of eight healthy individuals. One-way ANOVA, Dunnett’s test. **(B)** Relative percentage of circ_0001806 in the cytoplasm and nucleus of CD3^+^ T cells, GAPDH as the cytoplasm control, and U6 as the nuclear control. Paired *t*-test was used to calculate *p*-values. **(C,D)** Induction of circ_0001806 in peripheral blood primary CD3^+^ T cells **(C)** and Jurkat T cells **(D)** after exposure to *C. neoformans* for 72 h, without opsonin. 1:10 represents MOI of infection (host cells: *C. n*). Data are presented as the mean ± SD of three independent experiments. ****P < 0.0001.

Next, to explore the role and functional mechanism of circ_0001806 in the host immune response against *C. neoformans* infection, the specific cell type expression pattern of circ_0001806 was first identified in healthy human peripheral immune cells. Three types of PBMCs from healthy donors were isolated using CD3, CD14, and CD19 microbeads, and the level of circ_0001806 in the three types of immune cells was then detected by real-time fluorescent quantitative PCR. The relative level of circ_0001806 in T cells was significantly higher than that in B cells and monocytes (Figure 3A). The different abundance types in the cytoplasm or nucleic region are associated with different biological processes (Wilkinson and Shyu, 2001). Therefore, we further analyzed the sublocalization of circ_0001806 within CD3^+^ T cells. The nuclear and cytoplasmic components of CD3^+^ T cells were isolated. Circ_0001806 was significantly enriched in the cytoplasm of T cells (Figure 3B). The induction of circ_0001806 in both CD3^+^T cells and Jurkat T cells after *C. neoformans* exposure was detected. Up to 24 h after infection, there was no obvious increase in the circ_0001806 level, in a manner independent of MOI, both in primary T cells and Jurkat T cells. After 24 h of exposure, circ_0001806 was significantly induced in a time-dependent manner in both CD3^+^ T cells (1:1 and 1:10 group) and Jurkat T cells (1:5 group) (Figures 3C,D). These results showed robust circ_0001806 production in healthy CD3^+^T cells during *C. neoformans* exposure.

### Circ_0001806 Regulates Cell Apoptosis, G1S Arrest in Jurkat T Cells During *C. neoformans* Infection

**FIGURE 4:**
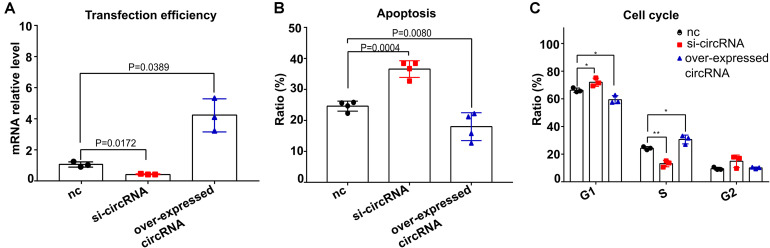
Reduced circ_0001806 levels on Jurkat T cells led to increased apoptosis and G1S arrest after exposure to *C. neoformans*. **(A)** Transfection efficiency of circ_0001806. One-way ANOVA, Dunnett’s test. **(B,C)** Apoptosis ratio **(B)** and cell cycle ratio **(C)** of the siRNA group, overexpressed circRNA group and control group of Jurkat T cells after exposure to *C. neoformans* were verified using flow cytometry. One-way ANOVA, Dunnett’s test Data are presented as the mean ± SD of three independent experiments. *P < 0.05, **P < 0.005.

Next, we evaluated how circ_0001806 is involved in the T cell response during *C. neoformans* exposure. Given the important role of cytokines in the T cell-mediated inflammatory response, the main T cell-related cytokines, including IL-10, IL-23, IL-4, GM-CSF, IFN-γ, IL-1β, IL-12p70, IL-13, IL-17A, IL-17F, IL-2, IL-21, IL-22, IL-28A, IL-5, IL-6, MIP-3α, TGF-β, TNF-α, and TNF-β, were measured using a Raybiotech cytokine microarray. No significant cytokine differences were detected (Supplementary Table 4). Considering the vital importance of apoptosis and the cell cycle in immune cells during infection (Parrino et al., 2007), we questioned whether circ_0001806 could impact other vital physiological functions. The impacts of circ_0001806 on cell apoptosis and the cell cycle were examined after *C. neoformans* exposure. Compared with the control group, the apoptosis level of Jurkat T cells in the siRNA-circ_0001806 group increased significantly after *C. neoformans* exposure, and the G1 phase was significantly prolonged with the S phase decreased (Figure 4), suggesting that there was a G1S phase arrest when the circ_0001806 level was reduced. To avoid the off-target possibility of si-circRNA, an overexpressed circ_0001806 plasmid was also constructed. Apoptosis decreased when transfected with the circ_0001806 plasmid after *C. neoformans* exposure. Additionally, a lower number of cells in the G1 phase and a higher number in the S phase were observed after overexpressing the level of circ_0001806 (Figure 4). Taken together, decreased circ_0001806 levels impaired T cell activity by promoting cell apoptosis and G1S arrest during *C. neoformans* exposure.

### Circ_0001806 Regulated Adrenomedullin (ADM) on Jurkat T Cells During *C. neoformans* Infection

**FIGURE 5:**
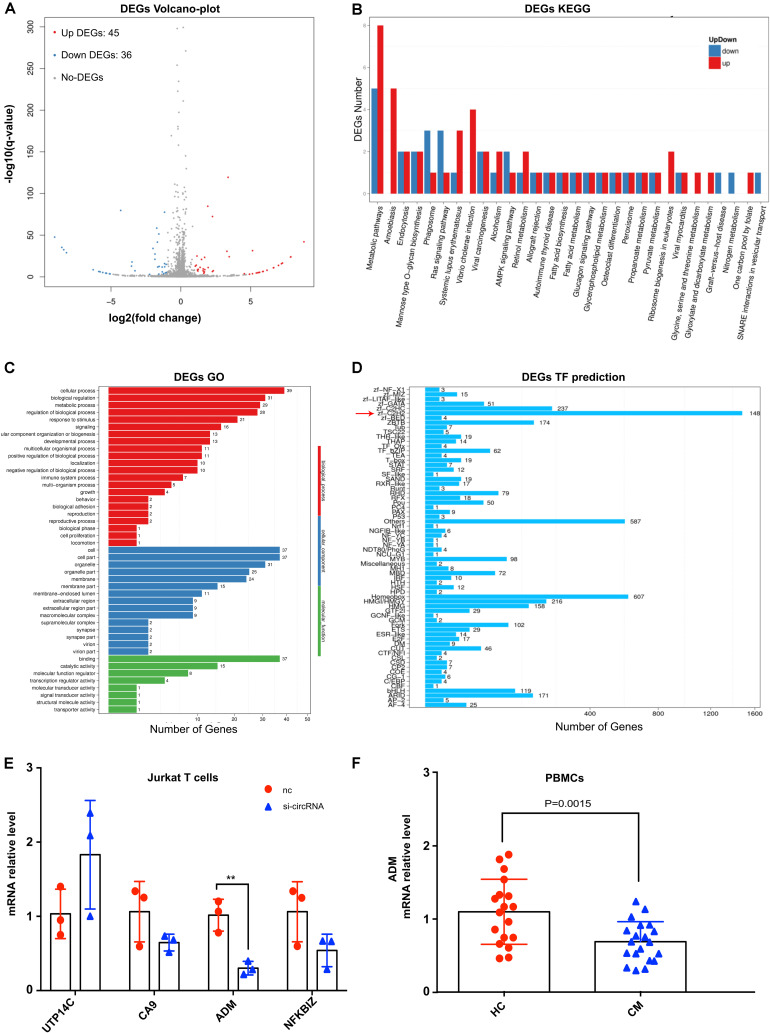
The impact of siRNA-circ_0001806 on the transcriptome of Jurkat T cells after *C. neoformans* exposure. **(A)** Volcano-plot of differentially expressed genes; red corresponds to upregulated, blue corresponds to downregulated, and gray corresponds to non-DEGs; *N* = 3 for each group; **(B)** KEGG analysis of differentially expressed genes; **(C)** GO analysis; red corresponds to biological processes (BPs), blue corresponds to cellular components (CCs), and green corresponds to molecular functions (MFs). **(D)** Classification of associated transcription factors. **(E)** Gene changes in Jurkat T cells after exposure to *C. neoformans* were verified by qRT-PCR. Data are shown as the mean ± SD from three independent experiments. **(F)** The mRNA level of ADM in PBMCs of twenty CM patients and eighteen healthy individuals; Unpaired *t*-test was used to calculate *p*-values, **P < 0.005.

To the best of our knowledge, for the first time, circRNA was identified to be associated with human fungal infection from the host side. To obtain a full map of transcriptome changes in the host mediated by circ_0001806 after *C. neoformans* exposure, high-throughput sequencing was performed in Jurkat T cells after transfection with siRNA-circ_0001806. Using *P* < 0.05 and fold change ≥2 as cut-off values, compared with the control, there were eighty-one significantly differentially expressed genes in total, with forty-five upregulated and thirty-six downregulated genes (Figure 5A). Listed in Supplementary Table 5, only thirteen genes were annotated, while most of the genes mediated by circ_0001806 were not annotated. For the KEGG analysis, the top three pathways mediated by circ_0001806 were Mannose type O-glycan biosynthesis, fatty acid biosynthesis and nitrogen metabolism (Figure 5B). O-glycan biosynthesis is a vital cellular process that is associated with T cell activation and homeostasis (Piller et al., 1988). Fatty acid biosynthesis is an attractive pathway, and the compounds of this pathway have been used as targets for anti-microbe drug research (Campbell and Cronan, 2001). The enrichment of these anti-microbe pathways mediated by circ_0001806 indicated the potential role of circ_0001806 in T cells during *C. neoformans* exposure. For the GO analysis, the top three biological processes (BPs) were cellular process, biological regulation and metabolic process; the top three cellular components (CCs) were cell, cell part and organelle; the top three molecular functions (MFs) were binding, catalytic activity and regulating molecular function (Figure 5C). The associated transcription factors were also predicted, and the most enriched was zf-C2H2 (Figure 5D). Then, four annotated genes were selected and verified by qRT-PCR. Compared with the control group, siRNA-circ_0001806 significantly downregulated the mRNA level of adrenomedullin (ADM) (Figure 5E). ADM is ubiquitously expressed in mucosal epithelial cells, such as in the skin and lungs, and participates in a broad range of cell processes, including apoptosis, proliferation and differentiation (Martínez-Herrero et al., 2012). An anti-microbe peptide feature of ADM in the host immune defense system has also been suggested over the past years (Zudaire et al., 2006). Therefore, we examined the involvement of ADM in CM patients. The mRNA level of ADM in PBMCs was nearly two-fold lower in CM patients than in healthy donors (Figure 5F), which is in line with the current evidence that cryptococcal patients exhibit cell immune defects (Elsegeiny et al., 2018).

### The Binding of Circ_0001806 With miRNA-126 Increased in Jurkat T Cells After *C. neoformans* Exposure

**FIGURE 6:**
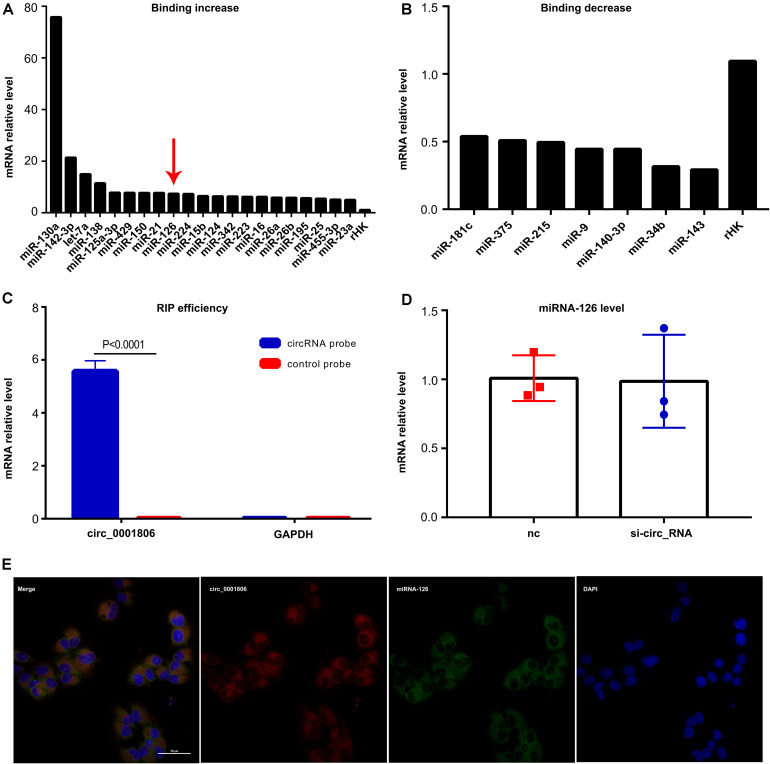
The binding of miRNA-126 with circ_0001806 increased in Jurkat T cells after *C. neoformans* exposure. **(A,B)** The increased **(A)** and decreased **(B)** binding of miRNAs with circ_0001806 after *C. neoformans* exposure was tested by RIP and microarray, and rHK was used as the internal control RNA. **(C)** RIP efficiency was verified by qRT-PCR. **(D)** miRNA-126 level after transfection with si-circ_0001806 in Jurkat T cells after *C. neoformans* exposure. **(E)** Colocalization of circ_0001806 (red) and miRNA-126 (green) was verified by FISH. Data are shown as the mean ± SD of three independent experiments. Unpaired *t*-test was used to calculate *p*-values.

Then, we aimed to determine the exact mechanism of how circ_0001806 regulates ADM, cell apoptosis and the cell cycle in T cells after exposure to *C. neoformans*. Circ_0001806 was overwhelmingly abundant in the cytoplasm of T cells (Figure 3B), suggesting that the functional mechanism was post-transcriptional regulation. It is widely known that circ_RNAs often act miRNA sponge (Liu et al., 2017). Therefore, RNA pull-down was performed in Jurkat T cells after *C. neoformans* exposure using a circ_0001806-specific probe, followed by microarray detection to determine the enrichment of the binding miRNAs. The binding of twenty-one miRNAs by circ_0001806 increased by more than five times, while the binding of seven miRNAs by circ_0001806 decreased by more than half (Figures 6A–C). This demonstrates circ_0001806 could serve as an efficient miRNA sponge. Overlap analysis was then performed by a full literature search which revealed that one candidate miRNA, miRNA-126, whose binding with circ_0001806 increased five times (Figure 6A, red arrow), can negatively regulate ADM expression in the stromal endothelium (Huang and Chu, 2014). If circ_0001806 acted by miRNA-126 binding, the total level of miRNA-126 would not be largely influenced. As shown in Figure 6D, no significant change in the level of miRNA-126 was observed in the group transfected with siRNA-circ_0001806. Moreover, FISH revealed colocalization of circ_0001806 and miRNA-126 in the cytoplasm (Figure 6E), which further supports circ_0001806 acts as a sponge for miRNA-126. Based on these results, we hypothesized that circ_0001806 could, via miRNA-126 binding, influence the function of ADM in T cells after *C. neoformans* exposure.

### Circ_0001806 Act as a miRNA-126 Sponge to Regulate Cell Apoptosis, the Cell Cycle and ADM in Jurkat T Cells After *C. neoformans* Infection

**FIGURE 7:**
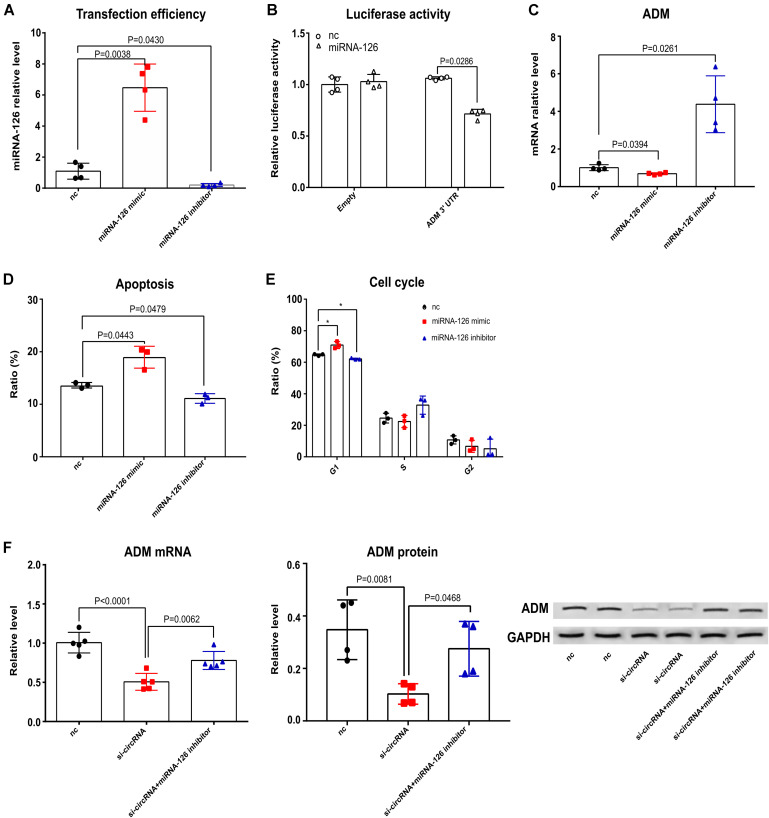
MiRNA-126 mimic had similar effects on Jurkat T cell apoptosis, cell cycle and ADM compared with reduced circ_0001806 levels after *C. neoformans* exposure. **(A)** Transfection efficiency of miRNA-126. One-way ANOVA, Dunnett’s test. **(B)** Luciferase activity analysis of ADM 3′UTR after miRNA-126 transfection. Unpaired *t*-test. **(C)** ADM expression in Jurkat T cells transfected with miRNA mimic or inhibitor after *C. neoformans* infection was tested by qRT-PCR. One-way ANOVA, Dunnett’s test. **(D,E)** Apoptosis ratio **(D)** and cell cycle **(E)** of the miRNA-126 mimic group, miRNA-126 inhibitor group and control group in Jurkat T cells after exposure to *C. neoformans* were verified using flow cytometry. One-way ANOVA, Dunnett’s test. **(F)** ADM mRNA (Left) and protein (Right) expression in Jurkat T cells after *C. neoformans* exposure when cotransfected with siRNA-circ_0001806 and miRNA-126 inhibitor. Data are presented as the mean ± SD of at least three independent experiments. One-way ANOVA, Dunnett’s test. **P < 0.005.

To address our hypothesis, we first tested the effects of miRNA-126 on Jurkat T cell apoptosis, the cell cycle and ADM after *C. neoformans* exposure. The luciferase reporter construct was developed by cloning the human ADM 3′-UTR sequence into the pMIR-Report construct. MiRNA-126 inhibited luciferase activity of the group that contained the 3′UTR of ADM (Figures 7A,B), demonstrating the binding of miRNA-126 to ADM. As shown in Figure 7C, the level of ADM was negatively regulated by miRNA-126 after *C. neoformans* exposure, which is in accordance with previous findings (Huang and Chu, 2014). Cell apoptosis and cell cycle, which were mediated by circ_0001806 after *C. neoformans* exposure in Jurkat T cells (as shown above), were evaluated in Jurkat cells transfected with miRNA-126 mimic or its inhibitor. The miRNA-126 mimic promoted T cell apoptosis and facilitated a switch to G1S arrest (Figures 7D,E), showing a similar phenotype to circ_0001806-deficient cells. Given that ADM is reported to regulate cell apoptosis and cell cycle (Sata et al., 2000; Ouafik et al., 2009), we postulated that ADM is the mediator through which circ_0001806 exerts effects on the T cell response. Notably, Jurkat T cells were co-transfected with siRNA-circ_0001806 and miRNA-126 inhibitor to examine whether the effects of circ_0001806 on ADM could be impaired by miRNA-126 inhibition. As shown in Figure 7F, the inhibitory role of deficient circ_0001806 on ADM expression in T cells after exposure to *C. neoformans* was partially suppressed by the miRNA-126 inhibitor at both the mRNA and protein levels. Taken together, these results suggest circ_0001806 functioned via miRNA-126 sponge.

## Discussion

CM in HIV-negative patients is a major issue, and its pathogenesis remains far from known (Williamson et al., 2017). Many risk factors are well known to be related to cryptococcal disease in HIV-negative individuals, especially immune defects such as the use of corticosteroids and immunobiology modifiers. In this report, it is noteworthy that of all the twenty CM patients, only three patients reported exposure to corticosteroid therapy, while the other patients were apparently immunocompetent, consistent with previous research that has shown that 10%–40% of cryptococcal patients are apparently immunocompetent (Pappas et al., 2001). To date, studies on host regulators during the immune response against *C. neoformans* focus on well-known cytokines and immune cells (Rohatgi and Pirofski, 2015). Little is known regarding the role of non-coding RNAs as host regulators in fungal infection. In this report, for the first time, we explored the involvement of the recently identified vital epigenetic regulation mechanism, circRNA, in the host response against *C. neoformans* exposure. Aberrant circRNA profiles have been identified in patients with infectious diseases, such as active pulmonary tuberculosis (Qian et al., 2018). In line with previously reported aberrant expression profiles of circRNAs during infectious disease, dysregulated circRNA profiles in HIV-negative CM patients were revealed in an exploratory analysis. Later, using a larger size validation cohort, we identified a significantly repressed circ_0001806 level (Figure 1).

Many circRNAs are inducible upon infection (Chen J. et al., 2019). Lan et al. (2016) found that a circRNA, mcircRasGEF1B, is induced upon microbial lipopolysaccharide stimulation to regulate ICAM-1 production. Here, a significant induction of circ_0001806 was observed after *C. neoformans* exposure both in primary CD3^+^T cells and Jurkat T cells in a time-dependent manner (Figures 3C,D). To the best of our knowledge, for the first time, circRNAs are reported to be induced in human immune cells upon fungal infection. However, it should be noted that given the universal regulatory roles of circRNAs in vital biological processes, circRNA_0001806 might also be induced by other pathogens including bacteria or viral components. Our data indicates that circ_0001806 is part of a protective host response to *C. neoformans* exposure, as a decrease in the circ_0001806 level was related to a higher fungal burden and shorter survival in the murine cryptococcosis model (Figure 2).

To date, studies of circRNA in immune cells predominantly focused on macrophages (Chen X. et al., 2019). The role of circRNA in T cells is less studied, although Wang et al. reported circular RNA100783 in CD28-related CD8^+^ T cell aging (Wang et al., 2015). In this report, we showed circ_0001806 to be involved in the T cell response in cryptococcosis in human. The increase in apoptosis and G1S arrest in T cells was regulated by decreased circ_0001806 levels upon *C. neoformans* exposure (Figures 4B,C). The decrease in circ_0001806 lead to higher fungal burden and shorter survival in mouse models, presumably by affecting the T cell apoptosis and proliferation. The inadequate cell immune activation, including T cell and classical macrophage activation, is widely known in HIV-negative cryptococcosis patients (Williamson et al., 2017). Given the protective role of circ_0001806 in T cells against fungal infection in this study, the decreased circ_0001806 in PBMCs from CM patients may also be due to the poor cell immunity in HIV-negative cryptococcosis (Figure 1).

ADM, as a multiregulatory molecule, could be induced by TNF-α under infection and inflammation and has been implicated in the host anti-microbe response by the interaction with complex receptor systems (Zudaire et al., 2006). The antimicrobial function of ADM is further demonstrated as the upregulation after TLR stimulation upon exposure to a wide range of pathogens both *in vitro* and *in vivo* (Allaker and Kapas, 2003). ADM is widely reported to inhibit apoptosis in human cells such as endothelial cells, keratinocytes, osteoblasts and fibroblasts (Albertin et al., 2003, Zhou et al., 2004; Uzan et al., 2008). The *in vitro* anti-apoptotic effect of ADM is regulated via mechanisms including MEK-ERK signaling (Yin et al., 2004; Uzan et al., 2008). In addition to apoptosis, ADM can also promote cell cycle via up-regulating cyclin D1 protein (Ouafik et al., 2009). Herein, ADM was positively regulated by circ_0001806 (Figure 5E) and was accompanied by changes in cell apoptosis and G1S arrest in Jurkat T cells (Figures 4B,C). T cells play a critical role in the clearance of *C. neoformans* infection, mediating a the balance of the Th1/Th2/Th17 (Jain et al., 2009; Murdock et al., 2014) and the protective regulation of NKT over γδ T cells (Kawakami, 2004). In addition to the indirect antifungal effects, human peripheral T cells have direct growth-inhibitory activity against *C. neoformans* (Levitz et al., 1994). This study indicates the indirect antifungal role of circ_0001806 in T cells via regulating anti-microbial ADM level. Moreover, CM patients also showed a decrease in the ADM mRNA level (Figure 5F). Considering the vital function of ADM in the host response against pathogens, the anti-microbe function of circ_0001806 during *C. neoformans* infection (Figures 2A,B) might be exerted by regulating the ADM levels in Jurkat T cells.

The most well-known mechanism of circRNA is its ability to function as an efficient miRNA sponge to target miRNA sites, such as the ciRS-7/miR-7 axis in neocortical and hippocampal neurons, to regulate gene expression (Hansen et al., 2013). Using RIP and literature overlap analysis, the miRNA-126 gene was postulated as a candidate circ_0001806 sponge. MiRNA-126 is located in an intron of Egfl7 and increasing evidence suggests an immune regulatory role for miRNA-126 in immune-related diseases (Qu et al., 2016). Inhibition of miRNA-126 resulted in increased activation of PU.1, which alters TH2 cell function by targeting GATA3 (Mattes et al., 2009). Despite its role in inflammatory diseases, little is known regarding miRNA-126 in infectious diseases except Zhao M et al. (Zhao et al., 2015) reported miRNA-126 decreased in the borna disease virus infected neonatal rats. The binding and colocalization of circ_0001806 to miRNA-126 by FISH suggests a potential regulatory function between circ_0001806 and miRNA-126. In addition, using RNA interfere technology, the effects of circRNA_0001806 can be partially disturbed by miRNA-126. In light of these results, we suggested miRNA-126 may serve as a sponge during circRNA_0001806 function.

The limitations of this work are the followings: (1) Because ADM levels were lower in the group co-transfected with circ_0001806 siRNA and miRNA-126 inhibitor than in the control group (Figure 7F), it is likely indicating that other regulatory mechanisms are also contributing to ADM function, (2) circRNAs may function in a wide range of mechanisms apart from miRNA sponge. Further studies on other potential regulatory mechanisms should also be explored, (3) Due to the quantities of PBMCs from CM patients, the protein level of ADM was not detected. 4) This study focused on the regulatory role of circ_0001806 in cryptococcal infection. Future experiments aiming at the involvement of circ_0001806/miRNA-126/ADM/T cell dysregulation axis in cryptococcal meningitis specifically, might be performed to further address the biological function of circ_0001806 during infection.

**FIGURE 8:**
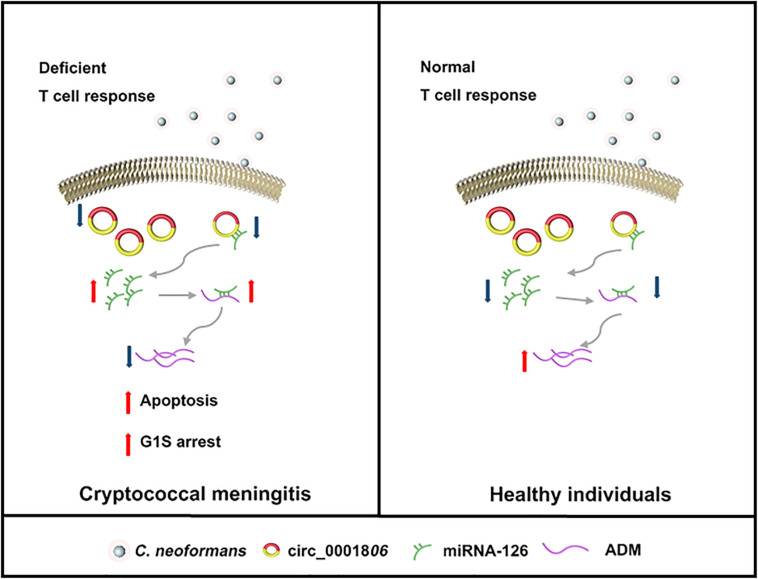
A graphic of the findings of this study. In healthy individuals with normal T cell response (Right), circRNA_0001806 is adequate enough to bind with miRNA-126. As a result, the free functional miRNA-126 is relatively low. The inhibitory role of miRNA-126 on ADM level is limited. Therefore, ADM is well produced and control the critical life processes such as apoptosis and cell cycle. In CM patients with deficient immune response (Left), decreased circRNA_0001806 causes higher cell apoptosis and G1S arrest, and lower ADM by miRNA-126 sponge.

A schematic of this mechanism is shown in Figure 8. In all, decreased circ_0001806 in CM patients was involved in the impaired T cell response against *C. neoformans* exposure by promoting cell apoptosis and G1S arrest, and ADM partially repressed the level via miRNA-126 sponge. Our study is the first to explore the epigenetic regulation mechanism in HIV-negative CM and could broaden the understanding of immunological pathogenesis in the host response to *C. neoformans* infection. As the involvement of circ_0001806 in cryptococcosis was investigated in HIV-negative CM patients, its role in cell immunity deficient CM individuals, such as organ transplant and HIV-positive should also be explored. Furthermore, we suggest that since lower ADM level in cryptococcal infection is associated with poor T cell response, ADM -targeted therapy might be a new strategy for cryptococcosis adjuvant therapy.

## Data Availability Statement

The original contributions presented in the study are publicly available. This data can be found here: https://www.ncbi.nlm.nih.gov/geo/GSE159601.

## Ethics Statement

The studies involving human participants were reviewed and approved by the Scientific Investigation Board of Second Military Medical University (Approval number: NSFC-31770161). The patients/participants provided their written informed consent to participate in this study. The animal study was reviewed and approved by the Scientific Investigation Board of Second Military Medical University (Approval number: NSFC-31770161).

## Author Contributions

LZ, WL, and WP designed the study. LZ, KZ, and WF performed the experiments. HL, YL, WJ, DH, XL, and LC contributed reagents or materials. LZ, KZ, WF, CC, WP, and WL prepared manuscript. All the authors contributed to the design, data analysis, manuscript preparation, and contributed to the article and approved the submitted version.

## Conflict of Interest

The authors declare that the research was conducted in the absence of any commercial or financial relationships that could be construed as a potential conflict of interest.

## Footnotes

1 www.circbase.org

## Supplementary Material

Supplementary Figure 1 The knock-down efficiency of siRNA circ_0001806 mouse counterpart.

Supplementary Figure 2 Representative graphs of apoptosis and the cell cycle of the siRNA group, the overexpressed circRNA group and the control group in Jurkat T cells after exposure to *C. neoformans*.

Supplementary Figure 3 Representative graph of apoptosis and cell cycle of the miRNA-126 mimic group, miRNA-126 inhibitor group and control group in Jurkat T cells after exposure to *C. neoformans*.

Supplementary Table 1 Clinical parameters of the cryptococcal meningitis patients.

Supplementary Table 2 Differially expressed circRNAs.

Supplementary Table 3 Sequence of circ_0001806 mouse counterpart siRNA.

Supplementary Table 4 Cytokine Concentration (pg/ml).

Supplementary Table 5 Transcriptome analysis of genes mediated by si-circRNA.

Supplementary Table 6 Primers for quantitative RT-PCR.

Supplementary Table 7 Sequence for siRNAs in cell transfection.

Supplementary Table 8-1 Probes for miRNA-pull down.

Supplementary Table 8-2 Primers for miRNA-pull down.

Supplementary Table 9 Probes for FISH.

Supplementary Table 10 Sequence for luciferase reporter assay.

Supplementary Table 11 Quality control of circRNA microarray.
