# Supplementary material for: CircRNA-1806 Decreases T Cell Apoptosis and Prolongs Survival of Mice After Cryptococcal Infection by Sponging miRNA-126
Source: Front Microbiol. 2020 Nov 13;11:596440. doi: 10.3389/fmicb.2020.596440 (PMC7691421; doi:10.3389/fmicb.2020.596440)
Supplement: Supplementary Figure 1 — The knock-down efficiency of siRNA circ_0001806 mouse counterpart. [file Data_Sheet_1.docx]

Supplementary Material

Table S1. Clinical parameters of the cryptococcal meningitis patients

**Supplementary Table 1 Clinical parameters of the cryptococcal meningitis patients**

| No. | Sex | Age | BMI | CD4% | Nutritional status | Previous history of diseases | Previous history of  immunosuppressive agent use | Titer in latex agglutination test (blood) |
| --- | --- | --- | --- | --- | --- | --- | --- | --- |
| P1 | F | 41 | 22.1 | 42 | Good | None | None | 1:40 |
| P2 | F | 21 | 19.3 | 32 | Medium | None | None | 1:1280 |
| P3 | M | 56 | 22.0 | 37 | Medium | None | None | 1:40 |
| P4 | M | 42 | 21.2 | 47 | Medium | Hospital-acquired pneumonia | Dexamethasone | 1:160 |
| P5 | M | 33 | 24.0 | 37 | Good | None | None | 1:160 |
| P6 | M | 35 | 19.6 | 39 | Medium | None | None | 1:640 |
| P7 | F | 56 | 20.2 | NA | Medium | Liver cancer | None | 1:40 |
| P8 | F | 31 | 19.2 | 32 | Medium | Hepatitis B | None | 1:40 |
| P9 | M | 21 | 21.3 | NA | Medium | Tuberculosis | None | 1:640 |
| P10 | M | 47 | 23.7 | 39 | Medium | Type 2 diabetes | None | 1:1280 |
| P11 | M | 51 | 20.1 | 35 | Poor | None | None | 1:2560 |
| P12 | M | 53 | 23.4 | 36 | Good | None | None | 1:640 |
| P13 | F | 47 | 17.7 | NA | Medium | None | None | 1:40 |
| P14 | F | 37 | 19.7 | 33 | Medium | Systemic lupus erythematosus | Methylprednisolone, dexamethasone | 1:40 |
| P15 | M | 44 | 19.3 | 49 | Medium | None | None | 1:160 |
| P16 | F | 54 | 20.5 | 32 | Medium | Acute rapidly progressive glomerulonephritis | Prednisone, methylprednisolone | 1:40 |
| P17 | F | 34 | 23.1 | NA | Good | None | None | 1:1280 |
| P18 | M | 50 | 24.0 | 33 | Good | None | None | 1:640 |
| P19 | M | 27 | 23.4 | NA | Good | None | None | 1:640 |
| P20 | M | 49 | 22.5 | NA | Good | None | None | 1:1280 |

BMI, Body mass index; M, male; NA: not available
